# High Diagnostic Accuracy of Long-Term Electrocardiogram Interpretation by General Practitioners

**DOI:** 10.1155/2024/6624344

**Published:** 2024-02-22

**Authors:** Jarle Jortveit, Miroslav Boskovic, Edvard Liljedahl Sandberg, Jonas Vegsundvåg, Sigrun Halvorsen

**Affiliations:** ^1^Department of Cardiology, Sorlandet Hospital, Arendal, Norway; ^2^Department of Cardiology, Sorlandet Hospital, Kristiansand, Norway; ^3^Department of Primary Health Care Services, Aalesund Municipality, Aalesund, Norway; ^4^Department of Cardiology, Oslo University Hospital Ullevaal, Oslo, Norway; ^5^Institute of Clinical Medicine, University of Oslo, Oslo, Norway

## Abstract

**Aims:**

Traditional long-term ECG monitoring systems have primarily been used by cardiologist. New remote and wearable easy-to-use devices have led to increased use of ECG recordings also outside cardiology clinics. The aims of this study were to assess the feasibility and diagnostic accuracy of interpretation of the one-lead ECG recordings from a patch ECG device (ECG247 Smart Heart Sensor system) by general practitioners (GP).

**Methods:**

Norwegian GPs were invited to digitally assess 10 long-term ECG recordings with different arrhythmias performed by the ECG247 Smart Heart Sensor system. For all ECG examinations, the presence/absence of different arrhythmias was registered.

**Results:**

A total of 40 GPs accepted the invitation and assessed all the 10 long-term ECG recordings. All the tests were assessed as interpretable by all the GPs. Arrhythmias (atrial fibrillation/flutter, supraventricular tachycardia, and ventricular tachycardia) were correctly identified in most cases, with sensitivity of 98% (95% CI 95-99%), specificity of 75% (95% CI 68-82%), and diagnostic accuracy of 89% (85-92%). Incorrect automatic system algorithm interpretations were rarely corrected by the GPs.

**Conclusion:**

GPs interpreted one-lead recordings by the ECG247 Smart Heart Sensor system with high diagnostic accuracy for common arrhythmias. However, in cases with rare arrhythmias, we recommend consulting a cardiologist to confirm the diagnosis before treatment is initiated. This trial is registered with NCT04700865.

## 1. Introduction

The electrocardiogram (ECG) is one of the most frequently used diagnostic tools in medicine and is a prerequisite for diagnosing cardiac arrhythmias. The ECG provides a snapshot of the heart's electrical signals, but long-term registration, defined as continuous ECG monitoring ≥ 24 hours, may be warranted to detect cardiac arrhythmias which may occur intermittently. Approximately 1,200 long-term ECG procedures per 100,000 inhabitants are performed in Norway every year, and the number of procedures increased by almost 70% over the last decade [1]. Performing and analyzing traditionally long-term ECG recordings are competence-, time-, and cost-consuming, and long-term ECG monitoring systems have primarily been used by cardiologists. However, previous studies have demonstrated interest among general practitioners (GPs) in increasing their knowledge in ECG interpretation and in examining/screening patients for atrial fibrillation (AF) and other heart rhythm disorders [2, 3].

New wearable easy-to-use devices have led to increased use of ECG recordings also outside cardiology clinics [4, 5]. Identifying basic arrhythmias requires only one ECG lead, which may simplify the interpretation by a GP with less experience in ECG assessment [6].

The aims of this study were to assess the feasibility and diagnostic accuracy of interpretation of long-term one-lead ECG recordings by GPs.

## 2. Materials and Methods

### 2.1. Study Design

This digital open-label nonrandomized diagnostic accuracy study was conducted as a substudy of the South-Norwegian self-screening for atrial fibrillation pilot trial at Sorlandet Hospital, Arendal, Norway. The main study included individuals ≥ 65 years old with at least one additional risk factor for stroke from the general population of Norway and demonstrated excellent feasibility for a fully digitalized self-screening procedure for atrial fibrillation (AF) [7]. This substudy was conducted and reported according to the Standards for Reporting Diagnostic Accuracy (STARD) recommendations [8].

### 2.2. Study Population

Norwegian GPs were invited to participate in the study between the 1^st^ of October 2022 and the 16^th^ of May 2023, by posting in closed social media groups for GPs and/or by direct invitations from colleagues. A total of 40 GPs in Norway participated in the study.

### 2.3. Diagnostic Device

**Figure 1:**
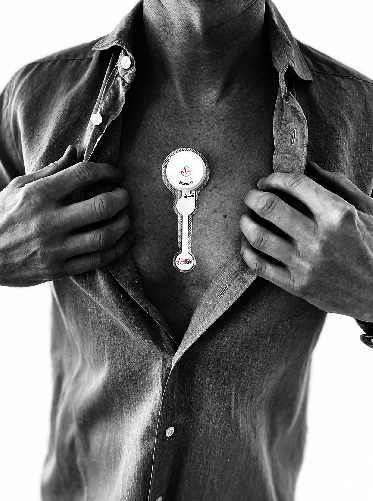
ECG247 Smart Heart Sensor system.

**Figure 2:**
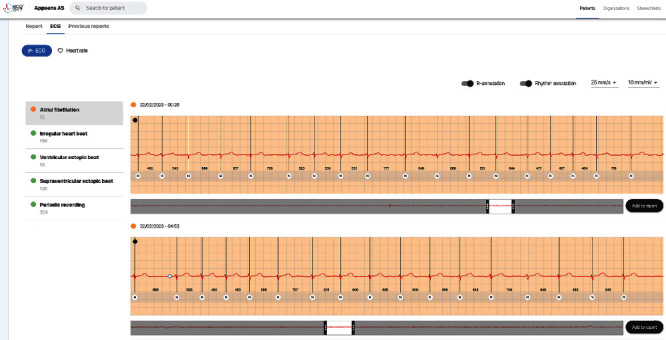
ECG247 web display of ECG recordings from the ECG247 Smart Heart Sensor system.

The long-term ECG recordings in the South-Norwegian self-screening for atrial fibrillation trial were performed by the ECG247 Smart Heart Sensor system (Appsens AS, Lillesand, Norway, http://www.ecg247.com/). The system consists of a disposable single lead ECG electrode patch, a reusable sensor, and a medical grade smartphone app with immediate transfer of ECG recordings to a secure medical back-end cloud service with automatic ECG interpretation (Figure 1) [9, 10]. All ECG recordings are automatically categorized according to severity and displayed in a web application (Figure 2). The ECG247 Smart Heart Sensor is CE certified according to the EU Medical Device Directive (93/42/EEC).

### 2.4. Study Procedure

**Figure 3:**
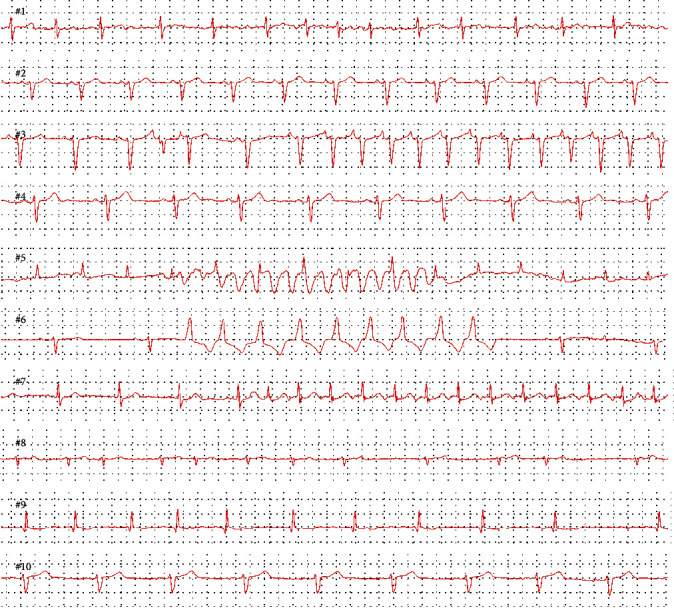
Examples of ECG recordings from the 10 test cases.

A total of 10 long-term ECG recordings were selected from the South-Norwegian self-screening for atrial fibrillation trial, consisting of a mixture of sinus rhythm and different common arrhythmias (Figure 3). Different arrhythmias (atrial fibrillation/flutter, supraventricular tachycardia, and ventricular tachycardia) were present in six of the ten selected long-term ECG recordings (Table 1). The median duration of the ECG recordings was 120 (61-207) hours. A total of 10 tests were chosen to have a representative sample of different ECGs and arrhythmias, as well as to limit the time spent on the study for the participating GPs.

All GPs were given digital access to these 10 long-term ECG examinations through the ECG247 web application. No clinical information was provided, but the system automatic algorithm interpretations were available as in an ordinary clinical setting.

All arrhythmias were verified by an external independent Data Monitoring Committee (DMC) consisting of an external independent cardiologist and a statistician.

For all ECG examinations, the presence or absence of the following arrhythmias were registered, of which GPs could select one or more per long-term ECG recording (yes or no): sinus rhythm, atrial fibrillation/flutter (AF) > 30 sec, supraventricular tachycardia (SVT) > 15 sec, ventricular tachycardia (VT) (>4 beats), and pause (≥4 sec).

After completing the ECG interpretations, all GPs were invited to answer a digital questionnaire focusing usability of the ECG247 web application. A system usability score (SUS) > 68 was defined as “acceptable” [11]. The survey was completely anonymous.

### 2.5. Outcomes

The feasibility endpoint was the proportion of long-term ECG recordings evaluated as interpretable by the GPs. The diagnostic accuracy endpoint was the proportion of tests with correct rhythm interpretation (different arrhythmias versus no arrhythmias (Table 1)) by the GPs. Finally, the usability of the ECG monitoring system was reported.

### 2.6. Reference Standard

Two independent cardiologists interpreted all long-term ECG recordings. No clinical information was available to these assessors.

### 2.7. Statistics

Continuous variables are presented as mean ± SD (standard deviation) or median (25^th^ and 75^th^ percentiles). Categorical variables are presented as numbers and percentages. Sensitivity, specificity, negative and positive predictive values, and accuracy are reported as percentages with 95% confidence intervals. The interpretations by cardiologists were used as the reference standard. The analyses were performed using STATA, version 17 (StataCorp, College Station, TX, USA). Summary statistics for diagnostic tests were conducted with the user-developed command “diagt.”

### 2.8. Ethics

The main study was approved by the Regional Committee for Medical and Health Research Ethics (REK 147963). All participants signed informed consent for study participation.

## 3. Results

A total of 40 Norwegian GPs accepted the invitation and participated in the study; 22 (55%) were male and 18 (45%) were females. Eight (20%) of the GPs were younger than 30 years, while 11 (28%) were >50 years old. The majority had ≥5 years' experience as a GP (*n* = 27, 68%), and 23 (59%) participants were certified as “specialist in general medicine.” Only 6 (15%) of the participants had clinical experience with traditional Holter equipment for long-term ECG recordings, while 33 GPs (83%) had experience with the ECG247 Smart Heart Sensor system.

All GPs assessed all 10 long-term ECG recordings (10 cases). All tests were assessed as interpretable by all GPs. The interpretations of the long-term ECG recordings by the automatic algorithm system, cardiologists, and the GPs are presented in Table 1.

Common arrhythmias, e.g., atrial fibrillation/flutter, supraventricular tachycardia, and ventricular tachycardia, were correctly identified in most cases. Incorrect automatic system algorithm interpretations were rarely corrected by the GPs. The diagnostic sensitivity, specificity, and accuracy for the overall detection of arrhythmia (i.e., atrial fibrillation/flutter, supraventricular tachycardia, and ventricular tachycardia) versus no arrhythmias are described in Table 2.

The usability of the ECG247 system was high with a mean system usability score (SUS) of 78 points.

## 4. Discussion

This study of 40 GPs assessing 10 long-term ECG recordings showed high feasibility of ECG interpretation by GPs. The diagnostic sensitivity was 98%, the diagnostic specificity was 75%, and the diagnostic accuracy was 89% for GP interpretation. Furthermore, the system usability score for the selected long-term patch ECG monitoring system (ECG247 Smart Heart Sensor) was satisfactory.

Several studies have investigated standard 12-lead ECG interpretation by GPs. Generally, the diagnostic accuracy has been shown to be high, and GPs are able to safely exclude significant arrhythmias [12, 13]. Similar, the diagnostic accuracy of handheld ECG devices in a community setting is high [14]. Although the interpretation of long-term ECG does not differ in principle from a standard 12-lead ECG or rhythm strips from handheld ECG devices, long-term ECG monitoring systems are mainly used by cardiologists. To our knowledge, this is the first study assessing GPs' ability to interpret long-term ECGs. This study supports previous findings regarding GPs' competence in ECG interpretation and indicates that heart rhythm assessment in many cases can be performed by GPs. Some advantages are faster diagnostic clarification and increased capacity at the cardiology clinics for more complicated cases.

Automatic algorithms may increase the sensitivity of GP interpretations [15, 16]. The agreement between the interpretations by the automatic algorithm and the GPs was high in this study. However, false positive algorithm interpretations may result in false positive interpretations by GPs such as in the case (#5) with a false positive episode of VT. Misinterpretations may lead to unnecessary further diagnostics and potentially harmful treatment. Telemedicine solutions and digital collaboration between GPs and cardiologists may contribute to increased diagnostic accuracy.

Many patients visit their GPs due to symptoms that may be caused by cardiac arrhythmias [17, 18]. Palpitations are a frequent indication for long-term ECG monitoring [1]. In patients without a history of heart disease or stroke, significant arrhythmias are rare [1]. New digital diagnostic tools enable immediate assessment by GPs. However, reliable interpretation by GPs is crucial for the safe implementation of such devices.

AF is the most common sustained cardiac rhythm disorder [19–23]. Due to the paroxysmal and often asymptomatic nature of AF, up to one-third of cases are undiagnosed [24, 25]. AF increases the risk of mortality, stroke, heart failure, cognitive impairment, hospital admissions, depression, and reduced quality of life [25]. The European Society of Cardiology (ESC) recommends screening for AF to be considered in people > 75 years of age and in all patients with an increased risk of stroke [25]. Recommendations on screening of high-risk individuals for AF and stroke are challenging for the specialist health service to handle. International guidelines are not followed today [1]. In patients with paroxysmal AF, AF will often be missed by a single ECG recording, while long-term ECG monitoring improves the detection rate [26, 27]. However, long-term continuous-ECG-monitoring equipment that is suitable, affordable, and sufficiently easy to use for screening purposes in larger populations has limited availability [28]. New patch devices like the Norwegian ECG247 Smart Heart Sensor system may enable self-screening for AF [7]. However, this will require committed GPs who can interpret the tests and start treatment according to guidelines [25].

This study has several important limitations. It was not possible to determine the participation rate due to the open invitation posted in closed social media groups for Norwegian GPs. The mode of invitation to participate in the study might have also introduced a selection bias. Many of the participants had some previous experience with the long-term ECG system. The selection of long-term ECG recordings with different arrhythmias was determined by the authors in advance. A different sample would possibly have produced different results. The number of long-term ECGs to be assessed was limited because the GPs were not able to spend lots of time participating in the study. Unlike in real life, we requested respondents to choose “yes” or “no” of a selected number of arrhythmias without any clinical information. In clinical practice, a GP should always consider all patient information (i.e., symptoms, other heart diseases, drugs, etc.) whenever interpreting an ECG. The time spent for the interpretation of the long-term ECG recordings was not reported.

## 5. Conclusions

GPs were able to interpret long-term ECG recordings with high diagnostic accuracy for common arrhythmias. New inexpensive easy-to-use long-term ECG monitoring systems can be useful tools for GPs in daily practice. However, we recommend a low threshold for consulting a cardiologist for confirmation of arrhythmias.

## Figures and Tables

**Table 1:** Interpretation of long-term ECG recordings by the ECG247 automatic system, cardiologists, and general practitioners.

Case	Duration of the ECG recording (hours)	Automatic system algorithm interpretation	Interpretation by cardiologists	Interpretation by general practitioners (*n* = 40)									
				Sinus rhythm		Atrial fibrillation/flutter		Ventricular tachycardia		Supraventricular tachycardia		Pause	
				*n*		*n*		*n*		*n*		*n*	
1	168	Sinus rhythm and atrial fibrillation	Sinus rhythm with episodes of atrial fibrillation	32	80%	40	100%	1	3%	1	3%	2	5%
2	45	Sinus rhythm	Sinus rhythm	40	100%	0	0%	0	0%	3	8%	0	0%
3	95	Sinus rhythm and short run of supraventricular tachycardia	Sinus rhythm and short run of supraventricular tachycardia	39	98%	1	3%	1	3%	33	83%	2	5%
4	13	Sinus rhythm	Sinus rhythm	40	100%	0	0%	1	3%	0	0%	1	3%
5	235	Sinus rhythm and short run of ventricular tachycardia	Sinus rhythm	39	98%	1	3%	31	78%	1	3%	0	0%
6	290	Sinus rhythm and short run of ventricular tachycardia	Sinus rhythm and short run of ventricular tachycardia	39	98%	1	3%	38	95%	0	0%	1	3%
7	220	Sinus rhythm, supraventricular tachycardia, and atrial fibrillation	Sinus rhythm and supraventricular tachycardia	35	88%	7	18%	0	0%	34	85%	0	0%
8	143	Atrial fibrillation	Atrial fibrillation	5	13%	39	98%	1	3%	1	3%	0	0%
9	96	Sinus rhythm and atrial fibrillation/flutter	Sinus rhythm and atrial fibrillation/flutter	28	70%	35	88%	0	0%	1	3%	2	5%
10	49	Sinus rhythm	Sinus rhythm	39	98%	1	3%	0	0%	0	0%	1	3%

**Table 2:** Diagnostic utilities for detection of arrhythmias (all defined types^∗^) versus no arrhythmias of long-term ECG recordings by the automatic algorithm system and by general practitioners.

	Sensitivity		Specificity		Positive predictive value		Negative predictive value		Accuracy	
	Value	95% CI	Value	95% CI	Value	95% CI	Value	95% CI	Value	95% CI
Interpretation by the automatic algorithm system	100%	54-100%	75%	19-99%	86%	42-100%	100%	29-100%	90%	56-100%
Interpretation by general practitioners (GPs)	98%	95-99%	75%	68-82%	85%	81-89%	96%	91-99%	89%	85-92%

^∗^Atrial fibrillation/flutter, ventricular tachycardia, and supraventricular tachycardia.

## Data Availability

The data underlying this article will be shared on reasonable request to the corresponding author (JJ).
